# Genome Sequence of a *Proteus mirabilis* Strain Isolated from the Salivary Glands of Larval *Lucilia sericata*

**DOI:** 10.1128/genomeA.00672-16

**Published:** 2016-07-28

**Authors:** Ye Yuan, Yu Zhang, Shuhua Fu, Tawni L. Crippen, David K. Visi, M. Eric Benbow, Michael S. Allen, Jeffery K. Tomberlin, Sing-Hoi Sze, Aaron M. Tarone

**Affiliations:** a Department of Biochemistry and Biophysics, Texas A&M University, College Station, Texas, USA; b Department of Computer Science and Engineering, Texas A&M University, College Station, Texas, USA; c Southern Plains Agricultural Research Center, Agricultural Research Service, USDA, College Station, Texas, USA; d Department of Molecular and Medical Genetics, University of North Texas Health Science Center, Fort Worth, Texas, USA; e Department of Entomology and Department of Osteopathic Medical Specialties, Michigan State University, East Lansing, Michigan, USA; f Department of Entomology, Texas A&M University, College Station, Texas, USA

## Abstract

We announce a draft genome sequence of a *Proteus mirabilis* strain derived from *Lucilia sericata* salivary glands. This strain is demonstrated to attract and induce oviposition by *L. sericata*, a common blow fly important to medicine, agriculture, and forensics. The genome sequence will help dissect interkingdom communication between the species.

## GENOME ANNOUNCEMENT

*Proteus mirabilis* is a gut-commensal bacterium associated with human urinary tract infections (1–4) and is a model for cellular communication (5–8). It is found in association with rotting proteinaceous material (9–13), the blow fly *Lucilia sericata* (14) (a fly used in maggot therapy [15]), and other flies associated with decomposing animal remains and animal wounds (9, 13). There are several reasons to hypothesize a commensal relationship between these species. *P. mirabilis* is hypothesized to enhance maggot therapy (15). This enhancement is partially due to the production of antibiotic molecules (16, 17), which kill microbes that are effectively controlled in maggot therapy (15, 18, 19). This aligned microbial control suggests that the bacterium and fly are in competition with similar bacterial species. Concurrently, the flies do not appear to effectively control *P. mirabilis* (19). Further, *Proteus* species have been identified in salivary gland samples of *L. sericata* (14, 20), a relatively clean tissue and a major source of molecules contributing to molecular antibacterial activities important to maggot therapy (21–24). Finally, swarming signals associated with *P. mirabilis* have been linked to fly attraction and oviposition, making the species a model for interkingdom signaling between bacteria and insects (7), which might have implications for medical, forensic, and agricultural research with decomposer flies and for microbial ecology.

Here, we present a draft genomic sequence of *P. mirabilis*. Genomic DNA was isolated from a colony derived from maggot salivary glands of *L. sericata* third-instar larvae raised on beef liver (7). Sequencing was performed using an Ion Torrent Personal Genome Machine (Life Technologies, Carlsbad, CA) after preparation with a NEBNext fast DNA fragmentation library prep set. This produced approximately 1,880,512 short reads, with an average length of 219 bp, totaling 412 Mbp, resulting in approximately 104-fold coverage. A total of 113 contigs were assembled using the PATRIC assembly service (25), with an *N*_50_ of 202,584 bp. This strain is highly similar to previously sequenced *P. mirabilis* HI4320 (NCBI accession no. NC_010554) and BB2000 (NCBI accession NC_022000), being more similar to BB2000. Draft genome assemblies based on CONTIGuator (26) indicate 49 contigs unique to this strain, with 98.6% of the assembled nucleotides aligning to either of the reference genomes. These observations support a previous finding that strains from this species exhibit lineage specific indels (27, 28), suggesting a species with a core genome and various auxiliary genes. Two contigs were found to have plasmid identities of >99%.

The draft genome contigs consist of 3,953,708 bp, with 38.43% G+C content. A total of 3,678 genes and 3,586 coding sequences (CDSs) were identified by the NCBI Prokaryotic Genome Annotation Pipeline (29). Seven prophage regions were identified among contigs with PHAST (30), of which three regions are intact, three are incomplete, and one is questionably functional. One of the prophage sequences predicted to be active is located near *rfaL*, which has been shown to impact fly behavior (7). Strain-specific gene functions and phage insertions will be useful in dissecting the interactions between *L. sericata* and *P. mirabilis.*

### Nucleotide sequence accession numbers.

This whole-genome shotgun project has been deposited at DDBJ/ENA/GenBank under the accession no. LTBK00000000; this is version LTBK01000000.
